# Reverse genetics reveals single gene of every candidate on *Hybrid sterility, X Chromosome QTL 2* (*Hstx2*) are dispensable for spermatogenesis

**DOI:** 10.1038/s41598-020-65986-y

**Published:** 2020-06-03

**Authors:** Kento Morimoto, Koki Numata, Yoko Daitoku, Yuko Hamada, Keiko Kobayashi, Kanako Kato, Hayate Suzuki, Shinya Ayabe, Atsushi Yoshiki, Satoru Takahashi, Kazuya Murata, Seiya Mizuno, Fumihiro Sugiyama

**Affiliations:** 1 Laboratory Animal Science, Doctoral Program in Medical Sciences, University of Tsukuba, 1-1-1 Tennodai, Tsukuba Ibaraki, 305-8575 Japan; 2 Laboratory Animal Science, Bachelor of Medical Science, University of Tsukuba, 1-1-1 Tennodai, Tsukuba Ibaraki, 305-8575 Japan; 3 Department of Clinical Laboratories, University of Tsukuba Hospital, 2-1-1 Amakubo Tsukuba, Ibaraki, 305-8576 Japan; 4 Laboratory Animal Resource Center and Trans-border Medical Research Center, Faculty of Medicine, University of Tsukuba, 1-1-1 Tennodai, Tsukuba Ibaraki, 305-8575 Japan; 5 Developmental Engineering & Embryology Group Genetically Engineered Models and Services Charles River Laboratories Japan, Inc., 955 Kamibayashi, Ishioka Ibaraki, 315-0138 Japan; 6 Laboratory Animal Science, Doctoral Program in Biomedical Sciences, University of Tsukuba, 1-1-1 Tennodai, Tsukuba Ibaraki, 305-8575 Japan; 7 Experimental Animal Division, RIKEN BioResource Research Center, 3-1-1 Koyadai, Tsukuba Ibaraki, 305-0074 Japan

**Keywords:** Evolutionary genetics, Genetics

## Abstract

F1 hybrid progenies between related subspecies often show hybrid sterility (HS) or inviability. HS is caused by failure of meiotic chromosome synapsis and sex body formation in house mouse. Previous studies identified two HS critical genomic regions named *Hstx2* on Chr X and *Hst*1 on Chr 17 by murine forward genetic approaches. HS gene on *Hst1* was reported to be *Prdm9*. Intersubspecific polymorphisms of *Prdm9* induce HS in hybrids, and *Prdm9* null mutation leads to sterility in the inbred strain. However, HS gene on *Hstx*2 remains unknown. Here, using knock-out studies, we showed that HS candidate genes on *Hstx2* are not individually essential for spermatogenesis in B6 strain. We examined 12 genes on *Hstx2*: *Ctag2*, *49*3*0*4*47F04Rik*, *Mir743*, *Mir46*5*d*, *Mir4*6*5c-2*, *Mir465b-1*, *Mir465c-1*, *Mir465*, *Gm1140*, *Gm14692*, *4933436I01Rik*, and *Gm6812*. These genes were expressed in adult testes, and showed intersubspecific polymorphisms on expressed regions. This first reverse genetic approach to identify HS gene on *Hstx2* suggested that the loss of function of any one HS candidate gene does not cause complete sterility, unlike *Prdm9*. Thus, the mechanism(s) of HS by the HS gene on *Hstx2* might be different from that of *Prdm9*.

## Introduction

Postzygotic reproductive isolation between related subspecies often results in hybrid incompatibility such as hybrid sterility (HS) or inviability^1–4^. This HS is the barrier distinguishing related species by disturbing the gametogenesis in the hybrid. HS is widely observed around intersubspecific hybrids such as yeast, plants, insects, birds, and mammals^1,2,4^. In 1922, Haldane’s rule was reported as the observation that if only one sex of the hybrid is inviable or sterile, that sex is the heterogametic sex (XY or ZW)^5^. Additionally more than 80 years ago, Dobzhansky, T. (1937) and Muller, H. (1942) proposed a two-locus model called Dobzhansky-Muller incompatibility (hereafter DMI) hypothesis that postzygotic isolation arises from a negative epistatic interaction between incompatible alleles that evolved on two independent evolutionary lineages^6,7^. Previous studies showed a large effect of Chr X (Chr Z) on reproductive isolation, particularly on HS or hybrid inviability^8–11^. In the last 30 years, several HS genes and hybrid inviability genes were identified in budding yeast, thale cress, fruit fly, and house mouse^12–25^. In *Drosophila*, HS genes: *OdsH* and *Ovd*, and hybrid inviability gene: *Hmr* and locate on Chr X^13,14,16,21^. However in house mice, these orthologous genes have not reported. Although several researchers proposed to explain this heterogametic HS or inviability as being controlled by Chr X (Chr Z) in vertebrata^26–28^, the X-linked (Z-linked) causative gene for Haldane’s rule and DMI has remained unclear for over a century.

The two HS loci, *Hst1* and *Hstx2*, were found by murine forward genetics^29–32^. The first HS locus in mice, *Hst1* was identified as a polymorphic variant on Chr 17 between two laboratory strains, C57BL10/Sn and C6H/Di, both predominantly of *Mus musculus domesticus* (hereafter *Mmd)* origin^29^. To identify HS gene on *Hst1*, Forejt, J. *et al*. established HS murine recombinant inbred model using PWD/Ph (hereafter PWD) inbred strain derived from *Mus musculus musculus* (hereafter *Mmm*) subspecies and C57BL/6 (hereafter B6) inbred strain derived from *Mmd* subspecies^33,34^. PWD was established from a single pair of wild mice of the *Mmm* subspecies caught in 1972 in Central Bohemia, Czech Republic^35^. Both subspecies diverged from a common ancestor approximately 0.3 to 0.5 million years ago^36^. This model shows asymmetric HS, wherein (PWD × B6) F1 hybrid (mating PWD female with B6 male) shows sterility, while (B6 × PWD) F1 hybrid (mating B6 female with PWD male) shows semi-fertility^35,36^. To identify these HS loci, Gregorová, S. *et al*. also established intersubspecific chromosome substitution (consomic) strains C57BL/6-Chr#^PWD/Ph^/ForeJ carrying individual *Mmm* chromosomes or their parts on *Mmd* background^37^. The HS gene on *Hst1* was identified by the forward genetic approach as PR domain containing 9 (*Prdm9*)^24^, and later was shown to control meiotic recombination hotspots^38,39^. *Prdm9* encodes for histone H3 lysine 4 trimethyltransferase that controls the hotspots of DNA double strand breaks (DSBs) at meiotic prophase in spermatocytes. *Hstx2* was found out by consomic strains C57BL/6-Chr X^PWD/Ph^/ForeJ carrying *Mmm* Chr X or their parts on *Mmd* background^32^. The 4.7 Mb (Chr X:64.9 Mb-69.6 Mb, GRCm38) HS critical region on Chr X, *Hstx2*, was identified by quantitative trait locus (QTL) analysis of male fertility phenotypes in these consomic strains^32^. They revealed that HS is also controlled by *Hstx2* of PWD^32^.

Unlike the HS gene *Prdm9* on *Hst1*, the HS gene on *Hstx2* remains unknown. Identifying the molecular mechanism of HS controlled by Chr X can unravel the molecular mechanism of Haldane’s rule in vertebrata. Forward genetic approaches have a limit to narrow down to only 4.7 Mb *Hstx2* critical region, because *Hstx2* may lie on a recombination cold spot^40,41^. Hence, reverse genetic approach is required to identify the HS gene on *Hstx2*.

In a mouse model of intersubspecific hybrids between B6 and PWD, HS needs three factors, *Prdm9* heterozygosity (*Prdm9*^B6/PWD^), autosomal heterozygosity and the PWD allele of *Hstx2* (*Hstx2*^PWD^)^42^. One of HS mechanism model was explained by hybrid incompatibility between *Prdm9*^B6/PWD^ and autosomal heterozygosity without DMI at least in house mouse^43^. Minisatellite structure of the zinc finger array coding on DNA binding sites of PRDM9 has interspecific polymorphisms between B6 and PWD^43,44^. The binding sites were evolutionally eroded by DNA double-strand breaks determined by PRDM9 in each strain^43,44^. The affinity of PRDM9^B6^ to B6 autosomes and the affinity of PRDM9^PWD^ to PWD autosomes decreases with evolution, respectively^43,44^. This is because the evolutionary erosion of genomic DNA occurs independently in each mouse sub-strain. In hybrid, PRDM9^B6^-determined hotspots occur mostly on the PWD chromosome and *vice versa*^43,44^. The major meiotic consequences of asymmetric DSB hotspots result in unrepaired DNA DSBs, meiotic asynapsis and abnormal sex body formation, leading to disruption of sperm formation^43^. Nevertheless, only this model cannot explain the asymmetric HS because this meiotic arrest is also controlled by *Hstx2*. *Hstx2*^PWD^ is responsible for full sterility of (PWD × B6) F1 hybrid, while the B6 allele of *Hstx2* (*Hstx2*^B6^) attenuates fertility of (B6 × PWD) F1 hybrid^32^. However, how *Hstx2* regulates HS remains elusive. Therefore, we proposed three hypotheses of the role of *Hstx2*. First hypothesis is that the spermatogenic function of HS gene(s) on *Hstx2* is lost in (PWD × B6) F1 male but partially remains in (B6 × PWD) F1 male. Second hypothesis is that dominant-negative *Hstx2*^PWD^ impedes the spermatogenesis in hybrids. Third hypothesis is that *Hstx2*^B6^ rescues fertility of hybrids. Here, to verify whether *Hstx2* are essential for spermatogenesis in the first hypothesis, we aimed to delete HS gene on *Hstx2* in B6 mice. We hypothesised that HS gene on *Hstx2* may have similar features to *Prdm9*, such as expressing in adult testes^45^, having intersubspecific polymorphisms on expressed region between *Mmm* and *Mmd*^43,44^, explaining HS mechanism by single locus^43^ and inducing sterility by knock-out (KO) in B6 genetic background^45,46^. If our first hypothesis is correct, the HS gene on *Hstx2* KO B6 mice would be sterile. Therefore, we aimed to identify HS gene on *Hstx2* through KO study.

To identify HS candidate gene, we investigated the expression of 32 genes on *Hstx2* in adult B6 testes using databases. Next, we analysed intersubspecific polymorphisms on expressed regions between B6 and PWD, also PWK/Phj (*Mmm* inbred strain, hereafter, PWK) by past reports and public database.

## Results

### Twelve HS candidate genes were identified on *Hstx2*

**Figure 1:**
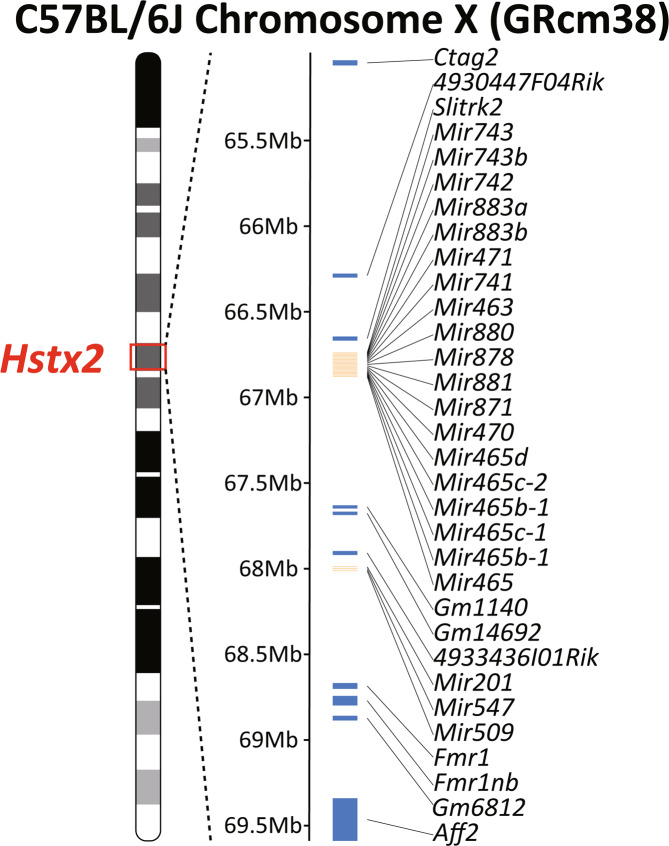
Gene map of the *Hstx2* critical region on Chr X. Gene map of the *Hstx2* critical region on Chr X (64.9Mb-69.6 Mb, GRCm38). *Hstx2* carries 10 protein-coding genes and 22 miRNA genes.

Male sterility of reciprocal hybrids between PWD and B6 is controlled by *Hstx2* (64.9 Mb-69.6 Mb, GRCm38)^32^, which carries 10 protein-coding genes and 22 microRNA (miRNA) genes (Fig. 1). As mentioned earlier, if our hypothesis is correct, the HS gene on *Hstx2* KO B6 mice must be sterile. To find out the HS gene from the 32 genes on *Hstx2*, we firstly screened them for expression in adult B6 testes. Testes expression was analysed using Fantom5 (http://fantom.gsc.riken.jp/5/), BioGPS (http://biogps.org/circadian/#goto=welcome) and miRbase (http://www.mirbase.org/), which revealed that of the 32 genes, 9 protein-coding genes: *Ctag2*, *4930447F04Rik*, *Gm1140*, *Gm14692*, *4933436I01Rik*, *Fmr1*, *Fmr1nb*, *Gm6812*, and *Aff2*, and all 22 miRNA genes were expressed in adult B6 testes (Table 1 and S1). The only one gene, *Slitrk2* showed no expression in adult B6 testes (Table 1). Next, we analysed intersubspecific polymorphism of the genes on *Hstx2*. We focused on intersubspecific polymorphism on expressed regions, which are the coding region in protein-coding alleles and mature miRNA region in miRNA alleles. Bhattacharyya *et al*. reported intersubspecific missense single nucleotide polymorphisms (SNPs) of HS candidate genes among B6, PWD, and PWK^32^ (Table 1). PWK is a *Mmm* inbred strain and closely related to PWD^47^. By comparison of the B6 allele of HS candidate genes with that of the PWD allele, *Ctag2*, *4930447F04Rik*, *Slitrk2*, *Mir743*, *4933436I01Rik*, *Fmr1nb*, and *Aff2* were found to carry missense SNPs between B6 and PWD. *Fmr1* does not carry any intersubspecific missense SNPs. We excluded *Slitrk2*, *Fmr1*, *Fmr1nb*, and *Aff2* on *Hstx2* as the targets, because these genes have already been reported as not being necessary for spermatogenesis in B6 male by KO studies^48–51^. Additionally, we targeted *Gm1140*, *Gm14692*, *Gm6812*, and *Mir465* cluster because *Gm1140*, *Gm14692*, *Gm6812*, *Mir465c-2*, *Mir465b-1*, *Mir465c-1*, and *Mir465* also have intersubspecific missense SNPs between B6 and PWK (Table S1). The next generation sequence (NGS) data on PWK was obtained from Sanger Institute Mouse Genome Project (http://www.sanger.ac.uk/science/data/mouse-genomes-project) and we aligned PWK and B6 reference genome (GRCm38). A very recent study reported that *Gm1140*, *Gm14692*, and *Gm6812* have intersubspecific missense SNPs between B6 and PWD, and *Mir465* cluster shows copy number polymorphism between B6 and PWD (and PWK)^40^. Therefore, we added these genes to the targets.

**Table 1:** Testis expression and intersubspecific missense SNPs between B6 and PWD of candidate genes on *Hstx2*.

Gene	testis expression	SNP position	B6	PWD	PWK
*Ctag2*	+	65,047,953	T	G	G
*4930447F04Rik*	+	66,303,564	A	C	C
*Slitrk2*	−	66,655,874	A	G	G
		66,656,111	A	G	G
*Mir743*	+	66,776,774	T	C	C
*493436I01Rik*	+	67,920,137	C	T	T
		67,920,143	T	G	G
		67,920,312	G	T	T
		67,920,431	T	A	A
		67,920,805	T	A	A
		67,920,818	C	T	T
		67,920,822	T	G	G
*Fmr1*	+	—	—	—	—
*Fmr1nb*	+	68,762,025	C	G	G
		68,769,064	T	A	A
*Aff2*	+	69,544,913	A	G	G
		69,830,745	C	T	T
		69,830,760	C	G	G
		69,830,782	A	T	T
		69,834,780	G	A	A

Taken together, we targeted 12 HS candidate genes, composed of six protein-coding genes, namely *Ctag2*, *4930447F04Rik*, *Gm1140*, *Gm14692*, *4933436I01Rik*, and *Gm6812*, and six miRNA genes, namely *Mir743*, *Mir465d*, *Mir465c-2*, *Mir465b-1*, *Mir465c-1*, and *Mir465*.

### Generation of eight HS candidate gene/cluster KO male mice by CRISPR/Cas9 system

To investigate whether each of the 12 HS candidate genes are essential for spermatogenesis, we generated each HS candidate gene KO mice using CRISPR/Cas9 system. We respectively designed two single guide RNA (sgRNA) sites, one upstream of the target gene and the other downstream of it on B6 genetic background in order to remove all genomic region. *Mir465d*, *Mir465c-2*, *Mir465b-1*, *Mir465c-1*, and *Mir465* are located on *Mir465* cluster, which is composed of 6 repeats (Fig. S1); as we could not design sgRNA for each gene of the *Mir465* cluster, we decided to knock out the complete *Mir465* cluster. To create each of the eight HS candidate gene/cluster KO mice, we performed the microinjection of *pX330*, the circular plasmid carrying sgRNA and Cas9 expression units, or the electroporation of sgRNA and Cas9 nuclease into B6 mouse eggs gained by mating with B6 male or *in vitro* fertilisation (IVF), and then each KO F0 founder were selected for KO allele, removing all genomic region of target gene. To confirm the CRISPR/Cas9 mutations, we performed genotyping using genomic PCR from mouse tail and then confirmed the KO allele. Thus, we generated each of the eight gene/cluster KO male founders (Table 2).

**Table 2:** Generation of each the HS candidate gene KO founders by CRISPR/Cas9 system.

Gene	Number of embryos		Number of pups			Number of founders			
	Injected	Transferred^a^	Newborn^b^	Weaning^c^	Birthrate^b/a^	Male	Female	Total^d^	Mutation efficiency^d/c^
*Ctag2*	83	80	31	31	38.8%	13	8	21	67.7%
*4930447F04Rik*	84	82	27	27	32.9%	13	7	20	74.1%
*Mir743*	155	146	41	38	28.1%	7	7	14	36.8%
*Mir465* cluster	75	72	26	24	36.1%	4	3	7	29.2%
*Gm1140*	266	249	38	36	15.3%	2	1	3	8.33%
*Gm14692*	100	98	18	16	18.4%	1	2	3	18.8%
*4933436I01Rik*	105	90	32	31	35.6%	10	7	17	54.8%
*Gm6812*	112	90	6	6	6.67%	1	2	3	50.0%

### Fertility and spermatogenesis of the eight HS candidate gene/cluster KO male mice

To investigate fertility of each candidate gene KO male founder, the sexually mature KO male F0 founder was mated with sexually mature B6 wild-type female. F0 founder have the possibility of mosaicism; therefore, these KO F0 male founder may not show full knock out of the gene in the testes. If KO allele was inherited to next generation, it would imply that the germ cell carried the KO allele and could be developed to fertilisable sperm. From mating test, each heterozygote KO female mice was born (Table 3). One of *4933496I01Rik* KO male founder generated wild-type female, suggesting that the founder was mosaicism. Except for the above, all other F1 female mice that were born from mating F0 male founders with female B6, showed the KO allele. Therefore, in our experiment, the male founders would not have shown mosaicism. These results suggested that the fertilisable sperm could have developed from the germ cell carrying the KO allele. Therefore, all eight KO male genotypes, 1 HS candidate gene/cluster per each, were fertile.

**Table 3:** Fertility of each the HS candidate gene KO male founders.

Gene	Founder number	Number of F1					
		Male			Female		
		Hemizygote	Wild-Type	Total	Heterozygote	Wild-Type	Total
*Ctag2*	#2	0	4	4	6	0	6
	#3	0	8	8	7	0	7
	#17	0	7	7	2	0	2
*4930447F04Rik*	#9	0	1	1	1	0	1
	#11	0	2	2	3	0	3
*Mir743*	#4	0	5	5	2	0	2
*Mir465* cluster	#1	0	5	5	3	0	3
	#14	0	6	6	0	0	0
	#16	0	2	2	1	0	1
*Gm1140*	#8	0	3	3	3	0	6
*Gm14692*	#46	0	8	8	9	0	9
*4933436I01Rik*	#7	0	7	7	5	0	5
	#8	0	6	6	4	0	4
	#20	0	8	8	3	5	8
*Gm6812*	#6	0	4	4	9	0	9

**Figure 2:**
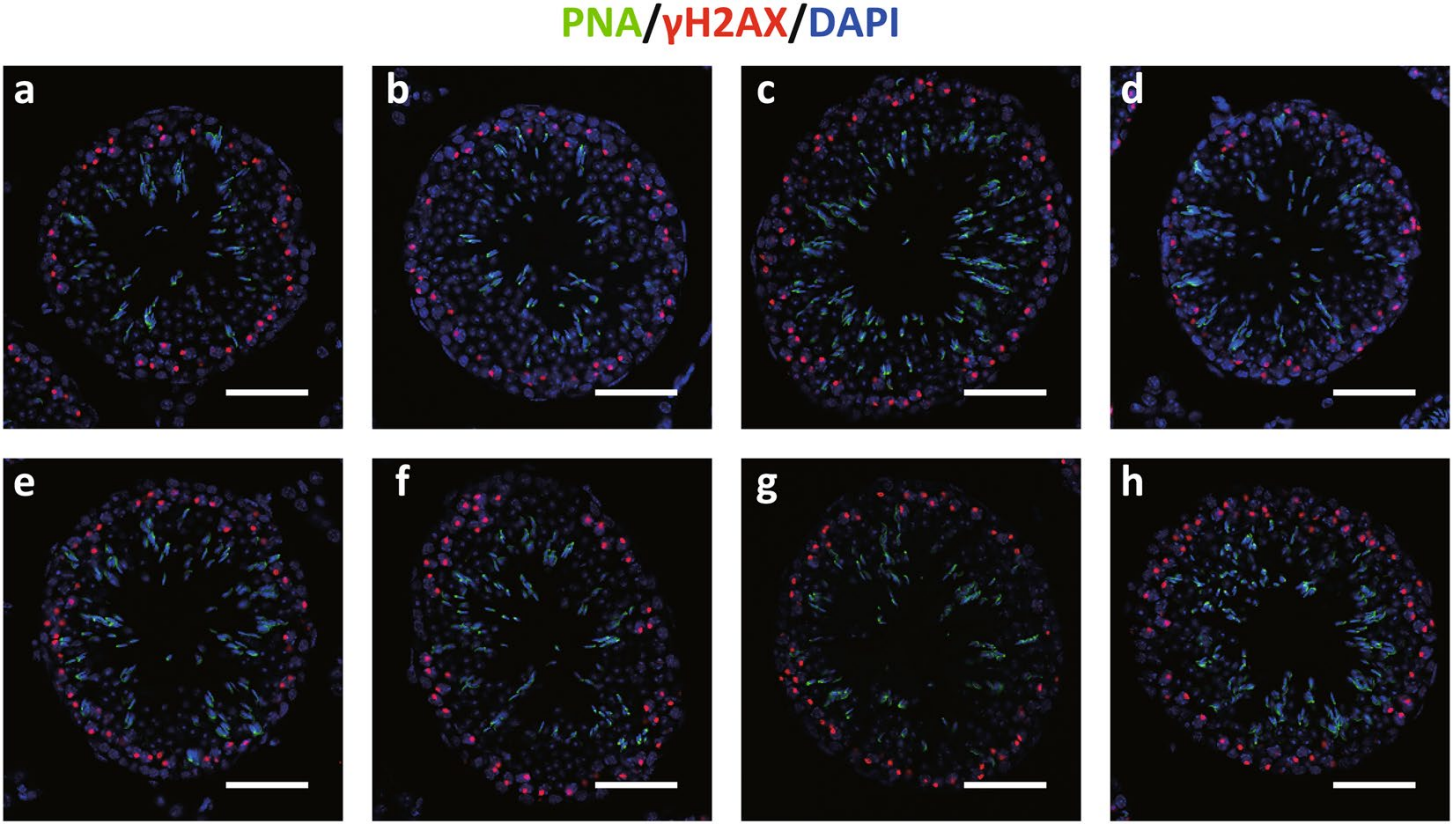
Histochemical analysis of the testis from each of the eight KO founders. PNA-LHC (green), γH2AX (red), and DAPI (blue) staining of the testis section of *Ctag2* (**a**), *4930447F04Rik* (**b**), *Mir743* (c), *Mir465 cluster* (**d**), *Gm1140* (**e**), *Gm14692* (**f**), *4933436I01Rik* (**g**), and *Gm6812* (**h**) KO founders. All show sperm in lumen of seminiferous tubule and sex body formation. Scale bar: 50 µm.

To investigate the spermatogenesis of each KO mice, histochemical analysis was performed. Peanut agglutinin (PNA) lectin histochemistry (LHC) is available as a marker of sperm. Immunohistochemistry (IHC) of phosphorylated form of the histone variant H2AX (γH2AX) is a marker of sex body on pachytene spermatocyte. IHC and PNA-LHC of testis section of each KO F0 founder showed sperm and sex body formation on these spermatocytes (Fig. 2). These results suggested that the eight candidate genes/cluster were not individually necessary for sperm formation at least in B6 genetic background.

## Discussion

Our reverse genetic approach revealed that the eight HS candidates, comprising six protein-coding genes, one miRNA gene and one miRNA cluster, were not individually necessary for spermatogenesis in B6 mice. These results exclude the possibility that the loss of function of one HS gene/cluster on *Hstx2* causes sterility, at least in B6 genetic background. In hybrid context between B6 and PWD, *Hstx2*^B6^ has semi-fertility but *Hstx2*^PWD^ fully lacks fertility^32^. Therefore, we hypothesised that *Hstx2*^B6^ and *Hstx2*^PWD^ are essential for spermatogenesis in both inbred strains and hybrids, and then the function of *Hstx2*^B6^ remains while that of *Hstx2*^PWD^ is disrupted in the hybrids. While we confirmed whether each HS candidate KO caused severe meiotic defects and meiotic failure in B6 mice, we did not assess more subtle phenotypes, such as a mere reduction in sperm count or motility. Therefore, these HS candidate KO males might show semi-fertility.

Previous studies identified two HS loci by forward genetic approaches using consomic strains^29,30^, and the HS gene on *Hst1* was identified as *Prdm9*^24^. However, these forward genetic approaches have a limit to narrow down 4.7 Mb (Chr X:64.9 Mb-69.6 Mb, GRCm38) of *Hstx2* critical region on PWD Chr X, because there may be recombination cold spot on *Hstx2*^40,41^. Recently, Lustyk, D. *et al*. reduced 4.7 Mb region of *Hstx2* to 2.7 Mb (Chr X:66.51 Mb-69.21 Mb) by backcross where SPO11-driven Cas9 nuclease was targeted by CRISPR to *Hstx2* interval in female meiotic prophase^40^. Nevertheless, this approach could not identify HS gene on *Hstx2*. Therefore, a reverse genetic approach is required to identify HS gene on *Hstx2*.

*Prdm9* is the only identified HS gene and is responsible for sterility phenotypes in male hybrids of house mouse^38,39,52^. Intersubspecific polymorphisms on the minisatellite cording for zinc-finger array of *Prdm9*, such as repeat variants and missense SNPs, and autosomal heterozygosity induce asymmetric DNA DSBs that cause sterility in hybrids^43,44^. Additionally, *Prdm9* is expressed in adult testes and its null mutation leads to sterility in the inbred strain^45,46^. Therefore, we hypothesised that HS gene on *Hstx2* may have similar features to *Prdm9*: intersubspecific polymorphisms of HS gene on *Hstx2* might cause HS, and the HS gene null mutation may lead to sterility on B6 background, as in the case of *Prdm9*. However, some hybrid with a sufficiently high recombination rate did not show sterility by *Prdm9* null mutation^46^, so that *Prdm9* is not essential for meiosis in male mice of every species. At least in inbred male, *Prdm9* is essential for spermatogenesis. In this study, we disregarded intersubspecific polymorphisms on intergenic and intron region. Therefore, another gene on *Hstx2* which has other polymorphisms such as SNPs on its regulatory region might be HS gene. Additionally, we added 4 genes/cluster: *Gm1140*, *Gm14692*, *Gm6812*, and *Mir465* cluster to the targets because these genes have intersubspecific missense SNPs between B6 and PWK. A very recent study reported that *Gm1140*, *Gm14692* and *Gm6812* have intersubspecific missense SNPs between B6 and PWD, and *Mir465* cluster have copy number polymorphism between B6 and PWD^40^. Therefore, this report supports our selecting.

However, in our study, none of the HS candidate genes on *Hstx2* was found to be essential for spermatogenesis on B6 genetic background. In our introduction, we proposed three hypotheses for the role of *Hstx2*. Based on our findings, we could partially exclude our first hypothesis, which was that the spermatogenic function of HS gene(s) on *Hstx2* is lost in (PWD × B6) F1 male, but partially remains in (B6 × PWD) F1 male. However, in this hypothesis, the polygenic effect is retained. On *Hstx2*, there are a few related and adjacent genes. The coding sequence of *Gm6812* is similar to that of *1700020N15Rik*, which is located nearby *Hstx2* (Chr X:69.9 Mb). The coding sequence of *Gm6812* has only one missense SNP compared with that of *1700020N15Rik* in both B6 and PWD mice. The coding sequence of *Gm1140* is also same as that of *Gm14692*. These genes might complement each other functionally. Besides these possibilities, recent study reported that KO of several genes on *Hstx2* resulted in arrested spermatogenesis in B6 mice^53^. The deletion of each *Mir741*, *Mir871*, and *Mir880* did not affect spermatogenesis, but deletion of 6 miRNAs, *Mir741*, *Mir463*, *Mir880*, *Mir878*, and *Mir871* disrupted spermatogenesis in B6 mice. Therefore, *Mir463* or *Mir878* might be essential for spermatogenesis, or polygene of these 6 miRNAs may have a critical role in spermatogenesis. The second hypothesis was that dominant-negative *Hstx2*^PWD^ impedes the spermatogenesis in hybrids. Nineteen miRNAs on *Hstx2* BAC transgenic mice carrying these autosomal genes show sterility^54^. Normally, these miRNAs are actively transcribed in spermatogonia and suppressed by meiotic sex chromosome inactivation in pachytene spermatocytes. In this BAC transgenic mice, these miRNAs are mis-expressed on pachytene, which induces spermatogenic defects in the BAC transgenic mice. Among the 19 miRNA genes, *Mir743* and the *Mir465* cluster showed intersubspecific polymorphisms between B6 and PWD. In our study, we generated *Mir743* single gene KO B6 mice and *Mir465* cluster KO B6 mice, which did not show male sterility. If the second hypothesis was correct, HS gene KO on *Hstx2* of PWD in (PWD × B6) F1 male would not show sterility. The third hypothesis was that *Hstx2*^B6^ rescues fertility of hybrids, while *Hstx2*^PWD^ cannot rescue HS. (PWD × B6) F1 show sterility caused by asymmetric DSBs^43^. Although the amount of asymmetric DSB in (B6 × PWD) F1 male spermatocyte is equivalent to that in (PWD × B6) F1 male, (B6 × PWD) F1 male shows semi-fertility^43^. This result suggests that HS in (B6 × PWD) F1 male is rescued without recovering the asymmetry of meiotic DSBs. Therefore, we propose another recovery mechanism of HS without repairing asymmetry of meiotic DSBs. We suspect that HS gene of PWD on *Hstx2* cannot rescue HS in (PWD × B6) F1 male, while HS gene of B6 on *Hstx2* can rescue HS in (B6 × PWD) F1 male, inducing asymmetric HS. If the third hypothesis was correct, HS gene KO on *Hstx2* of B6 in (B6 × PWD) F1 male would not show sterility.

In conclusion, we used a new approach to determine HS gene on *Hstx2* by reverse genetics, which revealed that *Ctag2*, *4930447F04Rik*, *Mir743*, *Mir465* cluster, *Gm1140*, *Gm14692*, *4933436I01Rik*, and *Gm6812* are not individually necessary for spermatogenesis in B6 mice. This result partially proved that the loss of function of one gene/cluster on *Hstx2* does not cause complete HS. Furthermore, the HS gene on *Hstx2* might cause HS by different mechanisms than that shown by *Prdm9*. Thus, our study contributes toward determination of the HS gene on *Hstx2* by at least excluding out one possibility.

## Methods

### Mice

C57BL/6 J (B6) and Jcl:CD1 (ICR) inbred strain were purchased from Charles River Laboratories (Yokohama, Japan) and CLEA Japan (Tokyo, Japan). All mice were housed in plastic cages under pathogen-free conditions in a room maintained at 23.5 °C ± 2.5 °C and 52.5% ± 12.5% relative humidity under a 14-h light:10-h dark cycle. Mice had free access to commercial chow (MF; Oriental Yeast, Tokyo, Japan) and filtered water. Animal experiments were carried out in a humane manner with approval from the Institutional Animal Experiment Committee of the University of Tsukuba in accordance with the Regulations for Animal Experiments of the University of Tsukuba and Fundamental Guidelines for Proper Conduct of Animal Experiments and Related Activities in Academic Research Institutions under the jurisdiction of the Ministry of Education, Culture, Sports, Science, and Technology of Japan.

### Immunohistochemistry of γH2AX and PNA lectin histochemistry

All mice were sacrificed over 10-week-old. After intraperitoneal (i.p.) injection of a fatal dose of pentobarbital, mice were transcardially perfused with phosphate-buffered saline (PBS) under anaesthesia. Testes, from which tunica albuginea were removed, were fixed overnight in 10%-Formaldehyde Neutral Buffer Solution (Nacalai Tesque, Kyoto, Japan) and then dehydrated in 70% ethanol at room temperature over 1 week. The fixed testes were embedded in paraffin and stored at room temperature until use. The testes were sectioned at a thickness of 6 µm, deparaffinised, immersed in 0.25% Triton X-100 in PBS and then the antigens were activated in Target Retrieval Solution (Dako, Santa Clara) for 10 min at 121 °C. After blocking the sections by the blocking solution [0.1% bovine serum albumin (BSA), 0.01% Tween20, 10% goat serum in PBS] for 60 min at room temperature, the sections were incubated with mouse IgG anti-γH2AX antibody (1:100, #05-636; Millipore, MA) overnight at 4 °C in a chamber with high humidity. After washing the sections twice using PBS, goat anti-mouse IgG Alexa Fluor 647 (1:200, #A28181; Invitrogen, CA) and PNA lectin (1:100, #L7381-1MG; SIGMA-ALDRICH, MO) were applied on the sections and incubated for 60 min at room temperature in a dark chamber with high humidity. The sections were washed twice with PBS and incubated with 4’,6-diamidino-2-phenylindole (DAPI) in PBS (1:500) for 10 min. After washing the sections using PBS, the sections were mounted with Prolong Gold antifade reagent with DAPI (Invitrogen). Images of seminiferous tubules were captured using a fluorescence microscope CCD camera (#BZ-X710; KEYENCE, Osaka, Japan) and processed using BZ-X analyzer software (KEYENCE).

### Sequencing and genotyping

Genomic DNA was extracted from the tails of 3-week-old mice. Genotyping PCR was performed using PrimeSTAR GXL DNA Polymerase (Takara Bio, Shiga, Japan) or AmpliTaq Gold DNA Polymerase (Applied Biosystems); the primers used for this are listed in Table S2.

### Generation of KO mice using CRISPR/Cas9 system

To remove all genomic region of each HS candidate gene, we respectively designed two sgRNAs, one whose target site was upstream of the target gene and the other was downstream of it on B6 genetic background. The sequences of the sgRNAs are listed in Table S3. To create KO mice, we performed microinjection or electroporation. The microinjection was performed according to previously reported method, with some modification^55^. Female B6 mice were i.p. injected with pregnant mare serum gonadotropin (PMSG) and human chorionic gonadotropin (hCG) with a 48-h interval, and mated with male B6 mice. The zygotes were collected from oviducts. Then, two *pX330* (circular, 5 ng/µl each), targeting two CRISPR sites of each HS candidate gene, was injected into the pronuclei according to standard protocols. The injected embryos (pronuclei stage) were then transferred into pseudopregnant ICR females. The electroporation was performed as reported previously, with some modification^56^. The sgRNA was synthesised and purified using the GeneArt Precision gRNA Synthesis Kit (Thermo Fisher Scientific, MA) and dissolved in Opti-MEM (Thermo Fisher Scientific). PMSG and hCG were i.p. injected into female B6 mice with 48-h interval, and unfertilised oocytes were collected from their oviducts. We then performed *in vitro* fertilisation with these oocytes and sperm from B6 mice according to standard protocols. Five hours later, two sgRNA (25 ng/µl each) targeting two CRISPR sites of each HS candidate gene, and GeneArt Platinum Cas9 Nuclease (100 ng/µl, Thermo Fisher Scientific) were electroporated into these zygotes using the NEPA 21 electroporator (Nepa Gene, Chiba, Japan). The poring pulse was set to: 225 V, 2 ms pulse width, 50 ms pulse interval, and + 4 pulse number. The transfer pulse was set to: 20 V, 50 ms pulse width, 50 ms pulse interval, and ± 5 pulse number (attenuation rate was set to 40%). After electroporation, the developed 2-cell embryos were transferred into the oviducts of pseudopregnant ICR females.

## Supplementary information

Supplementary information.
