# Supplementary material for: Reverse genetics reveals single gene of every candidate on Hybrid sterility, X Chromosome QTL 2 (Hstx2) are dispensable for spermatogenesis
Source: Sci Rep. 2020 Jun 3;10:9060. doi: 10.1038/s41598-020-65986-y (PMC7270182; doi:10.1038/s41598-020-65986-y)
Supplement: Supplementary file 1 — Supplementary information. [file 41598_2020_65986_MOESM1_ESM.pdf]

Supplementary information

Reverse genetics reveals single gene of every candidate on *Hybrid sterility, X Chromosome QTL 2* (*Hstx2*) are dispensable for spermatogenesis

Kento Morimoto[1], Koki Numata[2, 3], Yoko Daitoku[4], Yuko Hamada[4], Keiko Kobayashi[4,5], Kanako Kato[4], Hayate Suzuki[6], Shinya Ayabe[7], Atsushi Yoshiki[7], Satoru Takahashi[4], Kazuya Murata[4], Seiya Mizuno[4]*, Fumihiro Sugiyama[4]

[1]*Laboratory Animal Science, Doctoral Program in Biomedical Sciences, University of Tsukuba, 1-1-1 Tennodai, Tsukuba, Ibaraki, 305-8575, Japan.*

[2]*Laboratory Animal Science, Bachelor of Medical Science, University of Tsukuba, 1-1-1 Tennodai, Tsukuba, Ibaraki, 305-8575, Japan.*

[3]*Department of Clinical Laboratories, University of Tsukuba Hospital, 2-1-1 Amakubo, Tsukuba, Ibaraki, 305-8576, Japan.*

[4]*Laboratory Animal Resource Center and Trans-border Medical Research Center, Faculty of Medicine, University of Tsukuba, 1-1-1 Tennodai, Tsukuba, Ibaraki, 305-8575, Japan.*

[5]*Developmental Engineering & Embryology Group Genetically Engineered Models and Services Charles River Laboratories Japan, Inc., 955 Kamibayashi, Ishioka, Ibaraki, 315-0138, Japan*

[6]*Laboratory Animal Science, Doctoral Program in Biomedical Sciences, University of Tsukuba, 1-1-1 Tennodai, Tsukuba, Ibaraki, 305-8575, Japan.*

[7]*Experimental Animal Division, RIKEN BioResource Research Center, 3-1-1 Koyadai, Tsukuba, Ibaraki, 305-0074, Japan.*

*Corresponding author Mizuno Seiya: konezumi@md.tsukuba.ac.jp

A

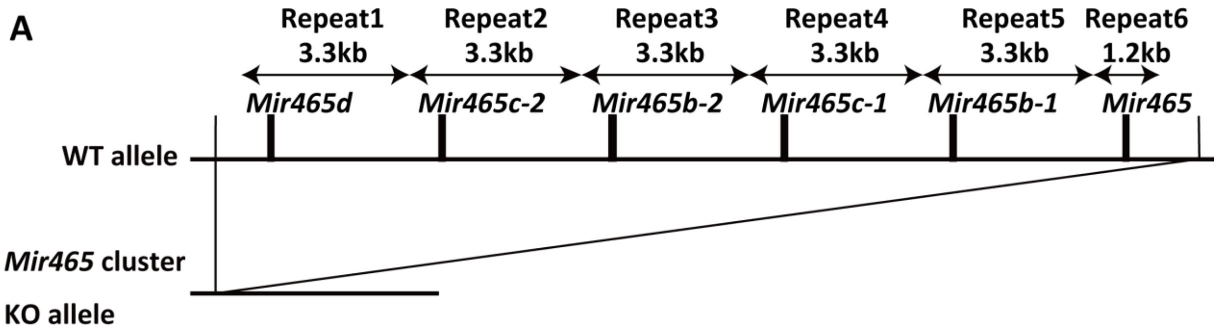

B

identity	Repeat1	Repeat2	Repeat3	Repeat4	Repeat5	Repeat6
Repeat1	-	88.9%	94.4%	88.9%	87.9%	85.2%
Repeat2	-	-	91.3%	100.0%	89.3%	85.2%
Repeat3	-	-	-	91.3%	93.3%	87.8%
Repeat4	-	-	-	-	89.3%	85.2%
Repeat5	-	-	-	-	-	87.8%
Repeat6	-	-	-	-	-	-

Figure S1. Six repeat sequence of *Mir465* cluster

(A) *Mir465* cluster has six repeat sequences. *Mir465* cluster KO mice have the complete *Mir465* cluster genomic region removed.

(B) Sequence similarity of the six repeats. Repeat2, composed of *Mir465c-2*, and Repeat4, composed of *Mir465c-1*, are completely same with each other.

Table S1. Testis expression and intersubspecific missense SNPs between B6 and PWK of non-reported genes on *Hstx2*

Gene	testis expression	SNP position	B6	PWK
Gm1140	+	67,690,875	G	T
		67,690,938	C	T
		67,693,489	T	C
		67,693,536	T	G
		67,693,537	T	G
Gm14692	+	67,695,723	A	C
		67,695,724	A	C
		67,695,771	A	G
Gm6812	+	68,892,645	A	C
Mir743b	+	-	-	-
Mir742	+	-	-	-
Mir883a	+	-	-	-
Mir883b	+	-	-	-
Mir471	+	-	-	-
Mir741	+	-	-	-
Mir463	+	-	-	-
Mir880	+	-	-	-
Mir878	+	-	-	-
Mir881	+	-	-	-
Mir871	+	-	-	-
Mir470	+	-	-	-
Mir465d	+	-	-	-
Mir465c-2	+	66,826,006	G	C
		66,826,012	C	T
		66,826,013	G	A
Mir465b-1	+	66,829,251	G	C
		66,829,257	C	T
Mir465c-1	+	66,832,568	G	C
		66,832,574	C	T
		66,832,576	G	A
Mir465b-1	+	66,835,813	G	C
		66,835,819	C	T
Mir465	+	66,839,109	G	A
Mir201	+	-	-	-
Mir547	+	-	-	-
Mir509	+	-	-	-

Table S2. The genotyping primers for the KO mice

Gene	Genotype	Primer1	Primer2	Band size (bp)
Ctag2	Wild-Type	CAACCACTGAGGATCCCCTGTGTTGCTTTTTGCTT	GGTCAGCTTGCCGATCTGGATGTGTGCTGTCCATC	447
	KO	CAACCACTGAGGATCCCCTGTGTTGCTTTTTGCTT	GGTCAGCTTGCCGATACTTTGCTTGCCTGGAAGAG	633
4930447F04Rik	Wild-Type	CAACCACTGAGGATCGGCTCAGCCATTAAGAGCAC	GGTCAGCTTGCCGATCCGTTGACATCACAAAGCAC	588
	KO	CAACCACTGAGGATCGGCTCAGCCATTAAGAGCAC	GGTCAGCTTGCCGATTGAAGCCATTTTGCATACTCA	527
Mir743	Wild-Type	CAGATTGGTGCCTGTCATGT	GGGCCTCAATCATGTCCTAA	360
	KO	CAGAGTGGTGCAGTGCAGTT	GGGCCTCAATCATGTCCTAA	442
Mir465 cluster	Wild-Type	TAGGGCAAGAAAGTGCGAGT	ATAGCCCCTGCCCTACACTT	939
	KO	TGCAATTTTAATCCTTTCATGGCACTGT	TGAAAACTTTGGCTTGGAAAAGGACTTC	1609
Gm1140	Wild-Type	TGCATCTCCTTCATGACCTG	AGACTGCATGGCCTATCTGG	456
	KO	TGCATCTCCTTCATGACCTG	AGCCATTTTTCCCCATAGAGTT	409
Gm14692	Wild-Type	AGACTACATGGCTTATCTCA	GATTGCCCCAAAATTGTGTT	541
	KO	GACCAAATCCTGGCTGAGAA	GATTGCCCCAAAATTGTGTT	550
4933436I01Rik	Wild-Type	CAACCACTGAGGATCTTTGAAGAGCAACCCCTCTG	GGTCAGCTTGCCGATCAGAGGGCCAAGTAGCTGTC	401
	KO	CAACCACTGAGGATCTTTGAAGAGCAACCCCTCTG	GGTCAGCTTGCCGATGCATGCATTTGCTTTTCTGA	390
Gm6812	Wild-Type	CAACCACTGAGGATCCGGGCAGGACAGTATGGAAGA	GGTCAGCTTGCCGATGGTGGTGGCACTTTTTGATT	356
	KO	CAACCACTGAGGATCCGGGCAGGACAGTATGGAAGA	GGTCAGCTTGCCGATTGGACTTTGTGGCAGGTACA	334

Table S3. Sequence of sgRNA for generation of KO mice

Gene	sgRNA1	sgRNA2
Ctag2	GCACTGTGACCCTCACAATG	TTAGCTAGTAAGAACCCACA
4930447F04Rik	GGGAACTCTAGAGTTGAACC	GTGCAACTGACATATGCAAG
Mir743	AGAAACTCATAGCCTACTGT	TTAATTCTCCAATCTATATG
Mir465 cluster	TATGAGGCTATGCTACTGCA	TGATTTATAAGTCCTCGGAA
Gm1140	CTTATCATACACATACCATG	TGGGATAGATTACAATGATG
Gm14692	TGGGATAGATTACAATGATG	GTTGTGAGCCAACCAGAATG
4933436I01Rik	AGTTACCCACCATTCACCAA	GAGAATAATTGCAAATCACA
Gm6812	TAGCCCACACTCCTCCTACA	GTCTAAGGTGAACCAGATGT
